# Jejunoduodenal intussusception caused by a solitary polyp in a woman with Peutz-Jeghers syndrome: a case report

**DOI:** 10.1186/1752-1947-8-13

**Published:** 2014-01-08

**Authors:** Ali Ozer, Pinar Sarkut, Ersin Ozturk, Tuncay Yilmazlar

**Affiliations:** 1 Department of General Surgery, Uludag University Faculty of Medicine, Görükle 16059, Bursa, Turkey

**Keywords:** Duodenum, Intussusception, Peutz-Jeghers syndrome

## Abstract

**Introduction:**

Peutz-Jeghers syndrome is a rare autosomal dominant disorder characterized by hamartomatous polyps and characteristic mucocutaneous pigmentation. The hamartomatous polyps of Peutz-Jeghers syndrome can cause intestinal occlusion, especially in the small intestine. Intussusception is seen frequently in children, but rarely in adults.

**Case presentation:**

We present the case of a 21-year-old female patient who was admitted to our emergency service with symptoms of ileus as a result of intussusception due to duodenal polyps. Radiological and endoscopic findings determined a jejunoduedonal intussusception. After an unsuccessful endoscopic attempt, a laparotomy was performed that revealed a polypoid mass originating from the fourth part of her duodenum, with intussusception of her proximal jejunum.

**Conclusion:**

Intussusception caused by Peutz-Jeghers syndrome is a rare diagnosis and is mostly jejunojejunal or jejunoileal. Despite the fact that a few duodenojejunal cases have been reported, this is to the best of our knowledge the first case of jejunoduedonal intussusception in a patient with Peutz-Jeghers syndrome to be described in the literature.

## Introduction

Peutz-Jeghers syndrome (PJS) is a rare autosomal dominant disorder characterized by hamartomatous polyps throughout the gastrointestinal tract and characteristic mucocutaneous pigmentation, primarily of the lips and oral and gingival mucosae. Polyps are found throughout the gastrointestinal tract but most are confined to the small bowel (60% to 90%) and the colon (50% to 64%). These polyps may also be found at extra-intestinal sites such as the gallbladder, bronchi, bladder and ureter [1].

The hamartomatous polyps of PJS have been reported to cause gastrointestinal bleeding, leading to iron deficiency anemia, in 14% of patients and recurrent intestinal obstructions due to the size of polyps in 43% of patients. However, adult intussusception is relatively rare; only 5% to 16% of cases are in adults, and they contribute to only 1% of all causes of intestinal obstruction [2].

The most seen types of intussusception are jejunojeunal, jejunoileal, ileoileal and ileocolic. However, the duodenal type of intussusception is rarely reported in the literature and likely depends on the retroperitoneal position of the duodenum in adults [3].

## Case presentation

**Figure 1:**
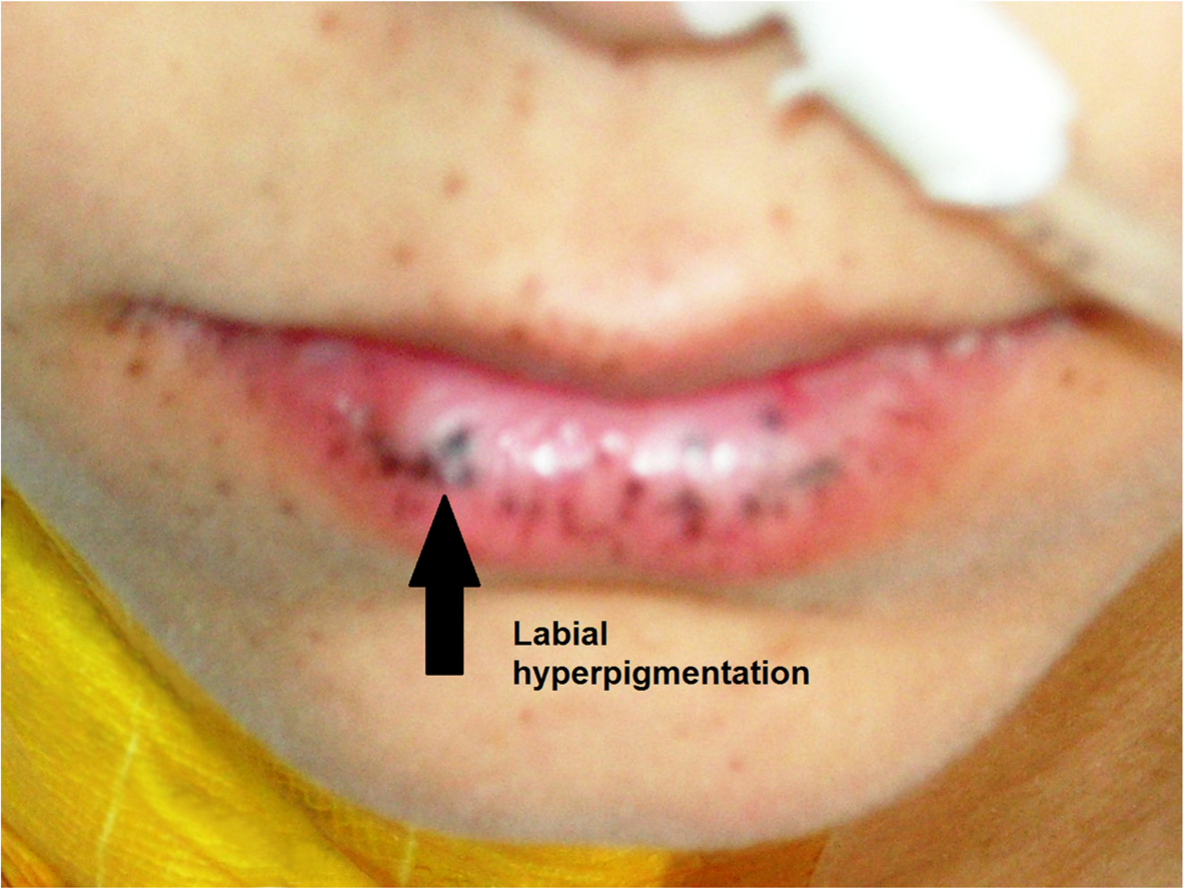
Labial hyperpigmentation.

A 21-year-old Turkish woman was admitted to our emergency service with complaints of abdominal pain and vomiting that had intensified over three days. This colicky abdominal pain was located in her right upper abdomen and was found to be non-radiating in nature. The pain became stronger after eating or drinking, and vomiting was found to relieve the pain for a while. Her upper abdomen was tender upon palpation, and guarding was significant in her right upper abdomen. Rebound tenderness or rigidity were not detected in our patient. In her right upper abdomen, an approximately 5cm-diameter mass was palpable and bowel sounds were significantly increased at this location. Mucocutaneous pigmentation was noted on her lips and oral mucosa (Figure 1).

Her vital signs were normal except for mild tachycardia (110 beats/min). Her leukocyte count was 13.1×109 cells/L, and her total and direct bilirubin counts were 4.0mg/dL and 2.9mg/dL, respectively. Her blood amylase level was slightly increased (230U/L). Except for the laboratory values noted, all other laboratory tests were deemed normal.

Our patient had been previously diagnosed with PJS 5 years before; however, she had no history of prior hospitalizations or surgery. Since the age of sixteen, our patient had experienced chronic abdominal pain and perioral hyperpigmentation. Because of her current complaints, further investigation had been performed at another hospital. Endoscopic and computed tomography (CT) enteroclysis findings had only shown a small solitary polyp in her proximal jejunum.

**Figure 2:**
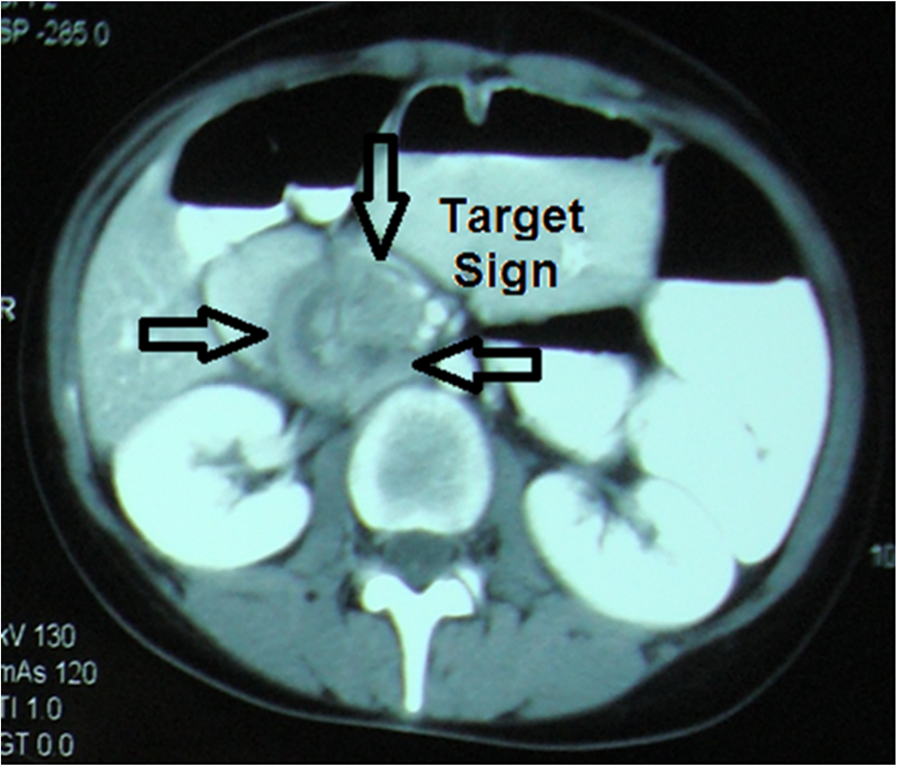
Computerized tomography shows target sign and duodenal obstruction (arrows).

Abdominal plain radiographs revealed distinct air-fluid levels at the level of her duodenum. An abdominal ultrasound revealed a target sign of invagination at the level of the right upper abdomen. Her stomach and the proximal part of her duodenum were dilated on contrast-enhanced CT, indicating the presence of an obstruction. Her intra- and extra-hepatic biliary tracts were found to be dilated, which was a result of the compression of invaginated jejunal segments on the papilla (Figure 2). The CT did not reveal any other polyps in her gastrointestinal tract. A colonoscopy had previously been performed in another clinic two months prior to the presenting symptoms, and the findings of that examination were normal.

We performed an esophagogastroduodenoscopy and found polyps in the fourth part of her duodenum. The invaginated jejunal fragments were also visualized. Reduction of the jejunal segments was attempted through gastroscopy, however this attempt was unsuccessful.

**Figure 3:**
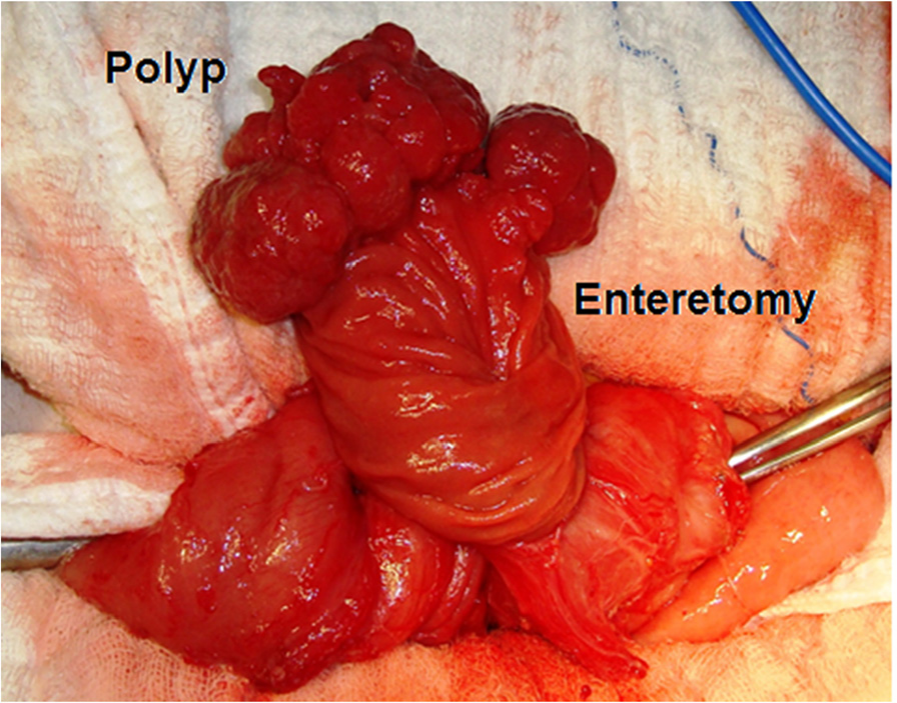
The polyp was excised through an enterotomy.

Technical difficulties with respect to the retroperitoneal position of her duodenum led us to carry out a laparotomy instead of a laparoscopy. Perioperative exposure revealed that the third and fourth parts of her duodenum were dilated. The Treitz ligament was loosened, and the invagination of the jejunal segments was gently reduced. We performed total excision of the polyps through an enterotomy at the last part of her duodenum (Figure 3). We subsequently made a thorough examination of her gastrointestinal tract; no other masses were palpated in the remaining parts of visualized gastrointestinal tract.

**Figure 4:**
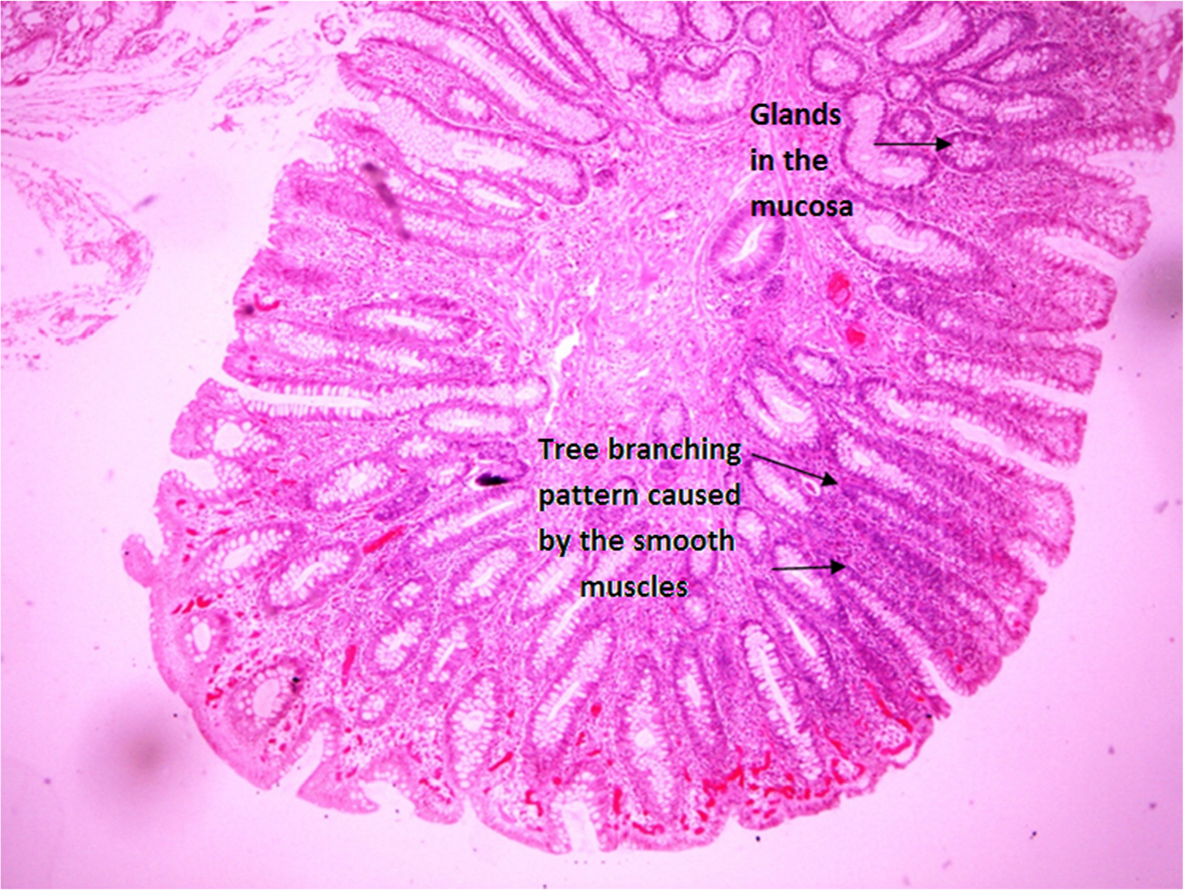
Hamartomatous polyp with branching muscular scaffolding and adenomatous changes.

Gross pathologic findings revealed an 8cm × 4cm × 3cm polypoid tissue mass. Histopathological examination revealed a hamartomatous polyp with adenomatous changes, without evidence of any malignancy. Immunohistochemical examination showed branching smooth muscle bands in the stroma of the polyp as typically seen in hamartomatous polyps of PJS (Figure 4).

Our patient had an uncomplicated postoperative course and was discharged home on postoperative day six. During the two-year follow-up period, our patient did not have any further episodes of intussusception.

## Discussion

PJS is a complex hereditary polyposis condition characterized by autosomal dominant inheritance, hamartomatous polyps of the gastrointestinal tract and characteristic mucocutaneous pigmentation. It is inherited in an autosomal dominant manner and is caused by a germline mutation in the *STK11* (*LKB1*) gene. The incidence has been estimated as one in 120,000 births. Fewer than 5% of patients with PJS lack the abnormal mucocutaneous melanotic pigmentation, and fewer than 5% of patients with the pigmentation have no PJ polyps [4].

A clinical diagnosis of PJS may be made when any one of the following conditions is present in a single individual: two or more histologically confirmed PJ polyps; any number of PJ polyps detected in one individual who has a family history of PJS in a close relative; characteristic mucocutaneous pigmentation in an individual who has a family history of PJS in a close relative; any number of PJ polyps in an individual who also has characteristic mucocutaneous pigmentation [1].

Intussusception occurs when one loop of bowel (intussusceptum) telescopes into an adjacent segment (intussuscipiens). This clinical presentation has been observed in 47% to 69% of adult patients with PJS and most of them were due to polyps located in the small intestine [3]. The majority of PJS intussusceptions reported in the literature are ileal or jejunal [2,5-7]. Colo-colonic intussusception is reported in only a few cases [8]. The duodenum is a particularly uncommon site for intussusception, since it lies in a fixed retroperitoneal position.

The diagnosis of intussusception caused by PJS should be based on a history of PJS and physical examination. Abdominal distention and local tenderness are the most frequent findings. We located a palpable mass in our patient but an abdominal mass has been noted in only 12.5% of cases [5]. Abdominal CT has been reported to be the most useful imaging modality. CT may help to delineate the precise location of polyps - differentiating between lead point and non-lead point intussusception is important in determining the appropriate treatment and has the potential to reduce the prevalence of unnecessary surgery [3].

Endoscopy has a distinct role in the diagnosis and treatment of intussusception. Endoscopic polypectomy and double-balloon enteroscopy are therapeutic options even in patients with a history of extensive abdominal surgery. Double-balloon enteroscopy may decrease the need for laparotomy in patients with PJS [9,10]. Furthermore, the use of this approach can lead to a healthier life and longer life expectancy by avoiding short bowel syndrome in the case of multiple intussusception. However, this technique has limitations for large solitary polyps and needs more experience. In our case, endoscopic reduction was unfortunately ineffective and we did not have the equipment to perform a double-balloon enteroscopy.

Endoscopic removal is the ideal method of treating a pedunculated polyp; however, when this is not possible, laparoscopy can be a safe and effective alternative for reduction of the intussusception and bowel resection [11,12]. In our patient, the polyp causing the intussusception was located in the fourth part of her duodenum. The retroperitoneal localization of her duodenum and invagination of her jejunum may have increased the risk of morbidity associated with laparoscopic management, especially in inexperienced hands. Therefore, we performed a laparotomy to reduce the intussusception and resect her bowel.

## Conclusion

Duodenal intussusception is a difficult situation because of its infrequency and nonspecific clinic presentation. Diagnosis is usually missed or delayed because of the patient’s nonspecific, chronic and recurrent symptoms. A thorough review of the patient’s history, physical examination, and radiological and endoscopic findings are critical in the case of jejunoduedonal intussusception, which is a rare form of an uncommon presentation.

## Consent

Written informed consent was obtained from the patient for publication of this case report and any accompanying images. A copy of the written consent is available for review by the Editor-in-Chief of this journal.

## Abbreviations

CT: Computed tomography; PJS: Peutz-Jeghers syndrome.

## Competing interests

The authors declare that they have no competing interests.

## Authors’ contributions

AO and PS analyzed and interpreted the patient data and researched the previous reports about this disease presentation. AO, PS and EO were the major contributors in writing the manuscript. EO performed the endoscopy to resolve the intussusception. TY performed an initial evaluation of the patient and was the primary surgeon in the operation. He also advised in evaluating the case characteristics and preparing the manuscript. All authors read and approved the final manuscript.
